# *In situ* production of branched glycerol dialkyl glycerol tetraethers in a great basin hot spring (USA)

**DOI:** 10.3389/fmicb.2013.00181

**Published:** 2013-07-09

**Authors:** Chuanlun L. Zhang, Jinxiang Wang, Jeremy A. Dodsworth, Amanda J. Williams, Chun Zhu, Kai-Uwe Hinrichs, Fengfeng Zheng, Brian P. Hedlund

**Affiliations:** ^1^Department of Marine Sciences, University of GeorgiaAthens, GA, USA; ^2^State Key Laboratory of Marine Geology, Tongji UniversityShanghai, China; ^3^School of Life Sciences, University of NevadaLas Vegas, NV, USA; ^4^Organic Geochemistry Group, Department of Geosciences, MARUM Center for Marine Environmental Sciences, University of BremenBremen, Germany

**Keywords:** branched GDGTs, hot spring, Great Basin, thermophilic bacteria

## Abstract

Branched glycerol dialkyl glycerol tetraethers (bGDGTs) are predominantly found in soils and peat bogs. In this study, we analyzed core (C)-bGDGTs after hydrolysis of polar fractions using liquid chromatography-atmospheric pressure chemical ionization-mass spectrometry and analyzed intact P-bGDGTs using total lipid extract (TLE) without hydrolysis by liquid chromatography-electrospray ionization-multiple stage mass spectrometry. Our results show multiple lines of evidence for the production of bGDGTs in sediments and cellulolytic enrichments in a hot spring (62–86°C) in the Great Basin (USA). First, *in situ* cellulolytic enrichment led to an increase in the relative abundance of hydrolysis-derived P-bGDGTs over their C-bGDGT counterparts. Second, the hydrolysis-derived P- and C-bGDGT profiles in the hot spring were different from those of the surrounding soil samples; in particular, a monoglycosidic bGDGT Ib containing 13,16-dimethyloctacosane and one cyclopentane moiety was detected in the TLE but it was undetectable in surrounding soil samples even after sample enrichments. Third, previously published 16S rRNA gene pyrotag analysis from the same lignocellulose samples demonstrated the enrichment of thermophiles, rather than mesophiles, and total bGDGT abundance in cellulolytic enrichments correlated with the relative abundance of 16S rRNA gene pyrotags from thermophilic bacteria in the phyla *Bacteroidetes, Dictyoglomi*, EM3, and OP9 (“Atribacteria”). These observations conclusively demonstrate the production of bGDGTs in this hot spring; however, the identity of organisms that produce bGDGTs in the geothermal environment remains unclear.

## Introduction

**Figure A1:**
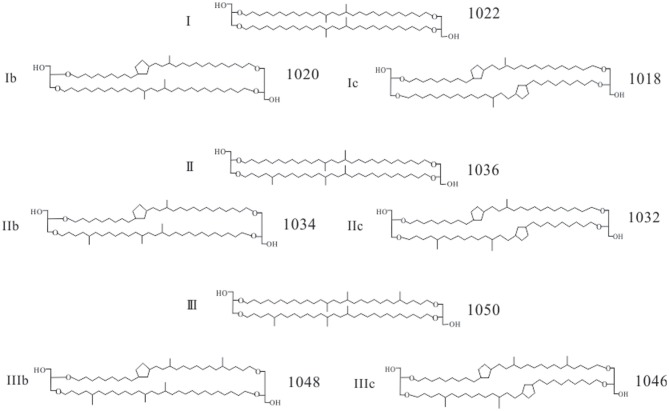
**Schematic structures of core branched glycerol dialkyl glycerol tetraethers (bGDGTs)**.

Recent advances in liquid chromatography-mass spectrometry (LC-MS) have significantly expanded our view of the occurrence of unique lipid biomarkers in the natural environment (Hopmans et al., 2000; Schouten et al., 2000, 2013; Sturt et al., 2004; Liu et al., 2012). Branched glycerol dialkyl glycerol tetraethers (bGDGTs; Figure A1) are unusual lipids that have been detected in a variety of natural settings using LC-MS, including soil and peat bogs (Hopmans et al., 2004; Weijers et al., 2006, 2007a; Peterse et al., 2009, 2010, 2012; Liu et al., 2010), lakes (Sinninghe Damsté et al., 2009; Tierney and Russell, 2009; Tierney et al., 2011; Sun et al., 2011; Wang et al., 2012), rivers and estuaries (Kim et al., 2010, 2012; Zhu et al., 2011; Zhang et al., 2012; Yang et al., 2013), and continental margin sediments (Weijers et al., 2007b,c; Schouten et al., 2008; Rueda et al., 2009; Bendle et al., 2010). While bGDGTs show clear structural similarities to known archaeal membrane lipids (Schouten et al., 2000), the source of bGDGTs was uncertain until recent studies demonstrated production of a single bGDGT, bGDGT I (see Figure A1), by two members of the phylum *Acidobacteria* (Sinninghe Damsté et al., 2011). Although other bGDGTs may possibly be produced by mesophilic *Acidobacteria* growing optimally in acidic and anoxic environments (Weijers et al., 2006; Sinninghe Damsté et al., 2011), this has yet to be determined unequivocally.

Despite their largely uncertain origin, bGDGT distributions tend to correlate with annual mean air temperature or soil pH (Weijers et al., 2007a; Peterse et al., 2009), thus having the potential to record paleocontinental temperatures (Weijers et al., 2007b,c; Ballantyne et al., 2010; Peterse et al., 2011, 2012; Weijers et al., 2011; Zhou et al., 2011) or soil pH (Weijers et al., 2007b; Tyler et al., 2010; Fawcett et al., 2011; Zhou et al., 2011). In combination with the archaeal biomarker crenarchaeol, bGDGTs are also used to estimate soil organic contribution in the marine environment (see review by Schouten et al., 2013).

bGDGTs have been reported from terrestrial hot springs in Yellowstone National Park, where the major source of bGDGTs was suggested to be soil runoff (Schouten et al., 2007). On the other hand, the presence of bGDGTs in mesophilic bacteria is recognized to be possibly a relict feature from thermophilic ancestors, as ether bonds as well as membrane-spanning core lipids have previously been reported in some thermophilic bacteria (Langworthy et al., 1983; DeRosa et al., 1988; Huber et al., 1992, 1996).

The Great Basin in western United States is an endorheic region with widely distributed geothermal activity. The hot springs of the Great Basin are characterized by low-inorganic energy yielding species such as ammonia, hydrogen sulfide, or hydrogen (Zhang et al., 2008), which are in contrast with more inorganic energy-rich geothermal systems fueled by subsurface volcanism (e.g., Yellowstone, Kamchatka, and Italy). The biological research in Great Basin hot springs has recently made important findings in lipid biomarker biogeochemistry and microbial carbon and nitrogen cycling processes (Pearson et al., 2004, 2008; Zhang et al., 2006, 2007; Huang et al., 2007; Costa et al., 2009; Dodsworth et al., 2011, 2013; Cole et al., 2013). Here we show multiple lines of evidence that bGDGTs are produced *in situ* in Great Boiling Spring (GBS) in the Great Basin.

## Materials and methods

### Sampling

**Figure 1:**
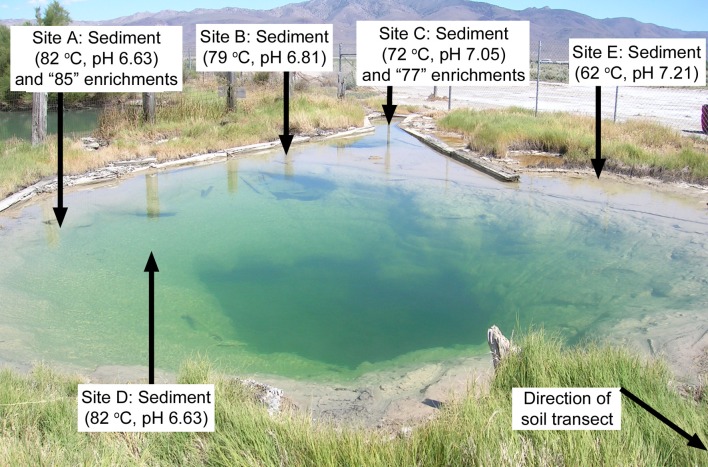
**Sites in GBS where sediments were collected and *in situ* cellulolytic enrichments were incubated**. The soil transect started at the edge of the hot spring at the lower right corner of the photo.

GBS is a large geothermal spring located in the US Great Basin near the town of Gerlach, Nevada [N40°39.689′ W119°21.968′; 9.15 m deep, 7.6 m diameter; described in Costa et al. (2009) and Dodsworth et al. (2011)]. GBS has a relatively well-mixed, oxic water column and a relatively uniform clay bottom composed primarily of smectite, illite, kaolinite, quartz, and zeolite (Costa et al., 2009). Sediment samples (top ~1 cm of sediment/water interface) were collected at five locations (Sites A, B, C, D, and E; Figure 1) in February 2010; at each location sediment was homogenized on site in a sterile pie tin. Subsamples of the sediment homogenate were separated into a 50-mL polypropylene tube for lipid analysis. Other subsamples were collected for a variety of other analyses, including identification of predominant minerals and 16 S rRNA gene pyrosequencing. Temperature and pH were measured at the precise location of sampling prior to sample collection using a LaMotte pH 5 meter (LaMotte, Chestertown, MD). The current paper focuses on analysis of GDGTs. The details of the mineralogy, 16S rRNA gene pyrosequencing, and field chemistry were reported in detail previously (Cole et al., 2013).

In addition, eight enrichments designed to stimulate growth of cellulolytic organisms were incubated *in situ*. Nylon bags (100 micron pore size, 10 × 10 cm) were filled with 20 g of either aspen shavings (AS) or ammonia fiber explosion (AFEX)-treated corn stover (CS). Bags were loaded into 20 × 12 × 5 cm polypropylene boxes punctured with ~100 0.5 cm holes to allow water exchange and incubated either suspended in spring water or buried ~1 cm deep in the sediment. The polypropylene boxes were anchored into the sediment or to structures adjacent to the spring by using stainless steel wire. Lignocellulose materials were incubated for 64 days (Figure 1, Site C; ~77°C) or 92 days (Figure 1, Site A; ~85°C). The difference in the incubation times was based on the time required to observe visual changes to the lignocellulose substrates that were consistent with cellulolysis. After incubation, the bags were removed, homogenized, and distributed into a sterile 50 mL polypropylene tube as described above for sediments. Details of lignocellulose degradation and 16S rRNA gene pyrosequencing were reported previously (Peacock et al., 2013).

The sample code for cellulolytic enrichments consists of three parameters, temperature (77°C or 85°C), cellulose substrate (A, aspen shavings; C, corn stover), and incubation environment (W, suspended in water; S, buried in sediment) as described earlier (Peacock et al., 2013). As described in Peacock et al. (2013), the temperature at each incubation site (within 0.5 meters of each lignocellulose enrichment) was tracked for the majority of the duration of the incubation using high-temperature iButtons (Maxim Integrated, San Jose, CA). During the time the temperature was tracked, temperatures ranged from 68°C to 82°C at site C (mean 77°C) and 74–88°C at site A (mean 85°C).

Finally, soil samples were collected at 10-, 20-, 30-, 50-, 150-, 200-, 300- and 500 cm distance from the edge (zero cm) of the spring (Figure 1) in order to provide a contrast in bGDGT profiles between the soil and the hot spring, which serves to evaluate possible soil contamination into the hot spring. This is a potential concern because of previous report of bGDGTs in soil next to hot springs in the Great Basin (Peterse et al., 2009). The soil temperature was determined by inserting a stainless steel temperature probe ~3 cm into the soil where the sample was collected. The soil pH was determined in the lab following a previously described procedure (Zhang et al., 2012).

All samples were frozen on dry ice in the field and stored at −80°C before analysis. DNA from each sample was extracted by using the FastDNA Spin Kit for Soil (MP Biomedicals, Solon, OH); raw data were presented in Cole et al. (2013) and Peacock et al. (2013).

### LC-MS analysis of polar bGDGTs (hydrolysis method)

Lipid extraction, fractionation, and separation of core (C)- and hydrolysis-derived polar (P)- bGDGTs followed a sonication method described in Zhang et al. (2012), in which the P-bGDGTs were calculated as the difference between the hydrolyzed and non-hydrolyzed polar fractions. The GDGTs were analyzed on an Agilent 1200 liquid chromatography equipped with an automatic injector coupled to QQQ 6460 MS and Mass Hunter LC-MS manager software using a procedure modified from Hopmans et al. (2004). Detection was performed using the Agilent 6460 triple-quadrupole spectrometer MS with an atmospheric pressure chemical ionization (APCI) ion source (Zhang et al., 2012). Separation of peaks was achieved using a Prevail Cyano column (2.1 mm×150 mm, 3 μm; Alltech Deerfield, IL, USA) maintained at a temperature of 40°C (Zhang et al., 2012). The detection limit of the LC-MS was 0.8 pg (Zhang et al., 2012).

### LC-MS analysis of intact polar bGDGTs (non-hydrolysis method)

While the hydrolysis method gives total abundance of all polar bGDGTs, it does not identify the types of polar bGDGTs. To identify specific head groups of the intact polar lipid bGDGTs, total lipid extracts (TLEs) were also analyzed by a reverse phase liquid chromatography-electrospray ionization-multiple stage mass spectrometry (RP-ESI-MS^n^) at University of Bremen, Germany (Zhu et al., in review). In brief, the analysis of TLEs was performed on a Dionex Ultimate 3000 ultra-high pressure liquid chromatograph (UHPLC) coupled to a Bruker maXis Ultra High Resolution orthogonal accelerated quadrupole—time-of-flight (qTOF) tandem MS/MS, equipped with an electrospray ionization source (ESI) in positive ionization mode (Bruker Daltonik, Bremen, Germany). Ether lipids were eluted through an ACE3 C_18_ column (3 μm, 2.1 × 150 mm; Advanced Chromatography Technologies Ltd., Aberdeen, Scotland), starting with 100% eluent A isocratically for 10 min, followed by a gradient to 24% eluent B in 5 min, and then to 65% eluent B in 55 min at a flow rate of 0.2 mL/min, where the eluent A was 100:0.04:0.10 of methanol/formic acid/14.8 M NH_3aq_ and B was 100:0.04:0.10 of 2-propanol/formic acid/14.8 M NH_3(aq)_. The column was washed with 90% eluent B for 10 min and subsequently re-equilibrated with 100% A for another 10 min. Ether lipids were scanned from *m/z* 100 to 2000 in a positive mode at a scan rate of 1 Hz with automated data-dependent fragmentation of the three most abundant ions. To ensure mass accuracy, an internal lock mass (*m/z* 922.0077) and tuning mixture solution (*m/z* 322.0481, 622.0290, 922.0098, 121.9906, 1521.9715, and 1821.9523) were infused directly into the ion source throughout a complete run and at the near end of the run, respectively. Lipids were detected as protonated [M+H]^+^, ammoniated [M+NH4]^+^, and sodiated [M+Na]^+^ molecular ions and identified by retention time, accurate masses (better than 1 ppm), and diagnostic fragments (Weijers et al., 2006; Liu et al., 2010).

### Statistical analyses

Mann-Whitney U tests and Wilcoxon signed rank tests were used as non-parametric alternatives to independent- and paired samples t-tests to explore relationships between bGDGT and experimental conditions. Linear regressions were calculated to quantify relationships between temperature and bGDGT fractions from sediment samples. These analyses were all calculated at the 0.05 level of significance.

Cluster analysis was performed on C-bGDGTs and hydrolysis-derived P-bGDGTs from soil and the hot spring samples using the base program in R 2.12.1. The relative abundances of C-bGDGTs and P-bGDGTs from all samples were imported into R and the Euclidean method was used to compute the distance matrix and generate a hierarchical clustering tree.

Spearman's rho, non-parametric correlation coefficients, were calculated to identify positive relationships between total bGDGTs (normalized to ng DNA) and relative abundance of phyla based on quality-filtered pyrotag sequence reads from cellulose enrichments. Analyses were completed for phyla that occurred at ≥1% relative abundance in one or more of the *in situ* cellulose enrichments. Subsequently, the same statistical framework was applied to individual Operational Taxonomic Units (OTUs) defined at 97% within phyla (Peacock et al., 2013) that were positively correlated with bGDGT abundance. Results are reported for 1-tailed significance.

## Results and discussion

### Abundance of C- and hydrolysis-derived P-bGDGTs

**Figure 2:**
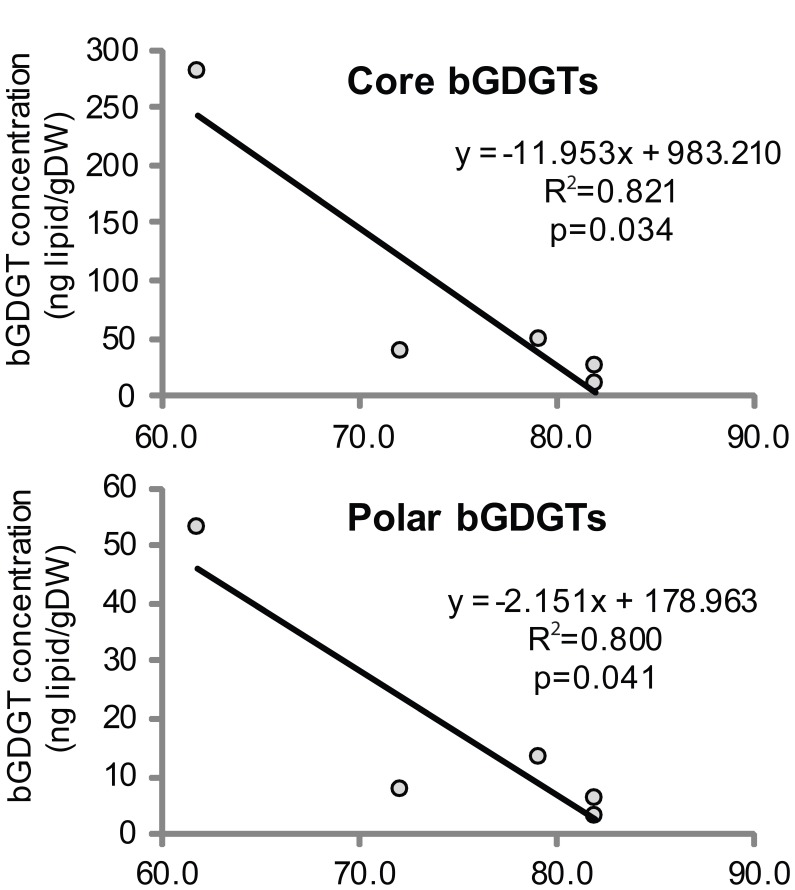
**Regression analyses indicated negative, linear relationships between the absolute abundance of C- and hydrolysis-derived P- bGDGTs in hot spring sediments and temperature (sig. < 0.05)**. bGDGT abundance was normalized to g dry mass of sample.

Sediment samples from the hot spring had C-bGDGTs ranging from 12 ng/g dry sediment to 280 ng/g dry sediment (Table 1), while P-bGDGTs were about 4-5-fold less abundant than C-bGDGTs (Table 1). Linear regression analyses indicated statistically significant, negative relationships between temperature and all bGDGT fractions (when normalized to gram dry sediment) from the hot spring sediments, with r^2^ values ranging from 0.80 to 0.82 (*p* < 0.05, Figure 2). The soil samples were also dominated by C-bGDGTs (up to 2–318 ng/g) with P-bGDGTs being less than 10 ng/g in five out of six samples (Table 1).

**Table 1:** **Core- and hydrolysis-derived polar bGDGTs from sediments, *in situ* cellulose enrichments in GBS, and soil samples along a transect from the hot spring (Figure 1)**.

			**bGDGTs (ng/g dry wt)**		
**Sample**	**Temp (°C)**	**pH**	**Core**	**Polar**	**% Polar**^**b**^
**SEDIMENTS**					
Site A	82.0	6.6	12	3	21
Site B	79.2	6.8	50	13	21
Site C	72.1	7.1	38	8	17
Site D	82.0	7.2	25	6	19
Site E	61.9	7.2	282	53	16
**ENRICHMENTS**^**a**^					
77AS	78.8	6.8	22	57	72
77AW	78.8	6.8	6	9	59
77CS	78.8	6.8	63	88	58
77CW	78.8	6.8	4	11	72
85AS	85.5	6.9	3	6	64
85AW	85.5	6.9	3	2	45
85CS	85.5	6.9	28	12	29
85CW	85.5	6.9	7	3	34
**SOILS**					
Soil-0 cm	NA^d^	8.2	NA	NA	NA
Soil-10 cm	33.6	7.8	149	8	5
Soil-20 cm	34.0	7.8	231	0	0
Soil-30 cm	30.9	7.4	319	7	2
Soil-50 cm	NA	NA	72	76	105
Soil-100 cm	31.7	8.2	NA	NA	NA
Soil-150 cm	NA	NA	2	0	6
Soil-200 cm	30.0	6.4	NA	NA	NA
Soil-300 cm	NA	NA	6	1	13
Soil-500 cm	30.5	9.2	NA	NA	NA
MAAT^c^	10.7				

a Enrichment sample codes consist of three parameters: average temperature (77°C or 85°C), cellulose substrate (A, aspen shavings; C, core stover), and incubation location (W, suspended in water; S, buried in sediment); temperature and pH listed are those measured at the time of sample collection.

b Hydrolysis-derived polar bGDGTs as a percentage of total (core + polar) bGDGTs.

c Mean annual air temperature at Gerlach, Nevada (http://www.wunderground.com/weather-forecast/US/NV/Gerlach.html).

d NA, not available.

bGDGTs were not detected in cellulosic substrates before incubation (data not shown) and *in situ* cellulolytic enrichments had C- and P-bGDGTs ranging from 2.0 ng/g solids to 88 ng/g solids. The cellulolytic enrichments had significantly higher percentages of P-bGDGTs than the hot spring sediments (Table 1; Mann-Whitney U test, *p* = 0.003), indicating enrichment of bGDGT-producing bacteria among cellulolytic consortia. Within the cellulolytic enrichments lower temperature sites (~77°C) had higher total bGDGT concentrations (C- plus P- bGDGTs) as compared to their corresponding higher temperature sites (Wilcoxon signed rank test, *p* = 0.068), which is consistent with the temperature relationships observed in the hot spring sediments (Figure 2). These observations suggest that organisms producing the bGDGTs tend to have higher biomass at lower temperatures (e.g., 60°C) in GBS.

Comparisons of paired cellulolytic enrichments showed that incubations within the sediment contained a higher absolute abundance of bGDGTs than their corresponding water column incubations (Wilcoxon test, *p* = 0.068), suggesting that anaerobic conditions favored bGDGT-producing organisms in this hot spring. Genetic data suggest the enrichments buried in the sediments were anaerobic. For example, the majority of 16S rRNA gene pyrotags described in Peacock et al. (2013) were strict anaerobes (e.g., dominant groups are Archaeoglobales, Thermotogales, Thermofilaceae, etc.). Paired comparisons of cellulosic enrichment substrates revealed that the absolute abundance of bGDGTs was elevated in corn stover as compared to aspen shavings (Wilcoxon test, *p* = 0.068), indicating a potential preference of bGDGT organisms for corn stover.

Schouten et al. (2007) first reported C-bGDGTs in hot springs, which accounted for up to 64% of total GDGTs. However, absolute concentrations of bGDGTs were not reported, precluding any comparisons with our data. Peterse et al. (2009), on the other hand, reported C-bGDGTs from geothermally heated soil near two hot springs in the Great Basin, which had the majority of C-bGDGTs (0.9–760 ng/g dry wt) in the same range as what is reported here (Table 1). More recently, two studies have reported the presence of bGDGTs associated with hydrothermal deposits in the mid-ocean ridges (Hu et al., 2012; Lincoln et al., 2013). Collectively, these observations suggest that bGDGTs can be produced at elevated temperatures, which may indicate a possible origin of bGDGTs in thermophilic bacteria.

### Composition of bGDGTs in sediments and enrichments

**Figure 3:**
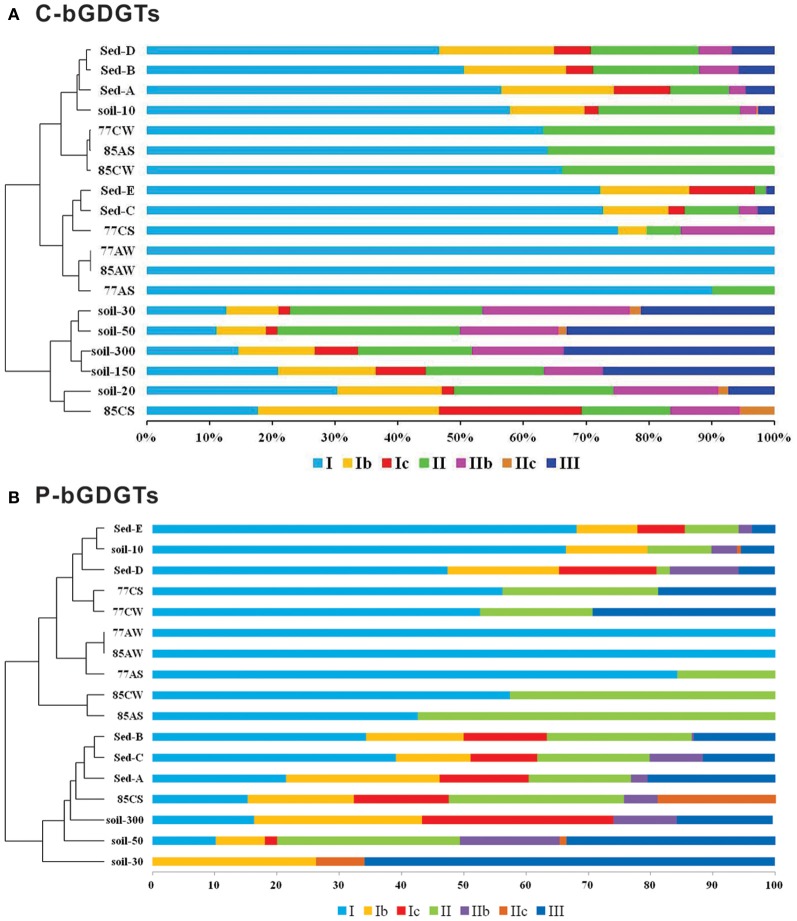
**Cluster analysis based on the relative abundance of C-bGDGTs (A) and hydrolysis-derived P-bGDGTs (B) from hot spring and soil samples, which distinguish the lipid profiles in hot spring sediments and cellulolytic enrichments from the majority of surrounding soil samples**.

Hot spring sediment samples contained bGDGT I, Ib, Ic, II, IIb, IIc, and III (Table 2). In most sediment samples, bGDGT I was the dominant bGDGT, but bGDGT Ib, Ic, II, and III were also present in significant amounts (>15% relative abundance) in one or more sediment samples. Cellulose enrichment generally led to simplification of bGDGT profiles, with bGDGT I comprising up to 100% of total bGDGTs in some enrichments with aspen shavings, and bGDGT II as the only other lipid detected in enrichments with aspen shavings. Enrichments with corn stover were more complex. bGDGT I was the dominant lipid in most corn stover enrichments, but bGDGT Ib, Ic, II, IIc, and III were also present in significant amounts (>15%) in one or more corn stover enrichments. The dominance of bGDGT I is consistent with the high relative abundance of bGDGT I in other hot spring environments (Schouten et al., 2007), in soils of warmer climate (Weijers et al., 2007a), and in some geothermally heated soils, although bGDGT I and II, with various degrees of cyclization, were present in roughly equal amounts in others (Peterse et al., 2009). The different composition of bGDGTs in the cellulolytic enrichments compared with those of the hot spring sediment samples (Sites A–E, Figures 1, 3), along with the increase in P-bGDGTs over C-bGDGTs, suggests that the enrichments stimulated growth of a distinct population of b-GDGT-producing thermophiles. This is also supported by cluster analysis based on relative abundance of C- and P-bGDGTs in soil samples as the majority of them were distinct from those collected from the hot spring environment (Figure 3).

**Table 2:** **Relative abundances of bGDGTs and bGDGTs-derived proxies for cellulolytic enrichments and natural samples from GBS**.

**Relative abundance of bGDGTs, normalized to 100% for core bGDGTs and for P-bGDGTs**															**bGDGT proxies**^**a**^							
	**I**		**Ib**		**Ic**		**II**		**IIb**		**IIc**		**III**		**MBT**		**CBT**		**T_**MBT/CBT**_ (°C)**		**pH_**CBT**_**	
**Sample**	**Core**	**Polar**	**Core**	**Polar**	**Core**	**Polar**	**Core**	**Polar**	**Core**	**Polar**	**Core**	**Polar**	**Core**	**Polar**	**Core**	**Polar**	**Core**	**Polar**	**Core**	**Polar**	**Core**	**Polar**
**SEDIMENTS**																						
Site A	56.5	21.5	17.9	24.6	9.0	14.3	9.4	16.4	2.6	2.7			4.6	20.5	0.83	0.60	0.51	0.14	30.9	22.8	7.4	8.4
Site B	50.5	34.3	16.3	15.7	4.3	13.3	16.8	23.3	6.3	0.3			5.7	13.1	0.71	0.63	0.47	0.56	25.1	20.4	7.5	7.3
Site C	72.8	39.1	10.4	12.0	2.6	10.7	8.6	18.0	3.0	8.6			2.7	11.5	0.86	0.62	0.78	0.44	29.4	20.7	6.7	7.6
Site D	46.6	47.4	18.4	17.9	5.8	15.6	17.2	2.2	5.3	11.0			6.8	5.8	0.71	0.81	0.43	0.24	25.3	32.2	7.6	8.1
Site E	72.3	68.1	14.2	9.8	10.5	7.6	1.7	8.6	0.2	2.2			1.2	3.7	0.97	0.86	0.71	0.81	35.7	29.1	6.9	6.6
**ENRICHMENT SAMPLES**																						
77AS	90.1	84.3					9.9	15.7														
77AW	100.0	100.0																				
77CS	75.0	56.2	4.6				5.4	25.0	14.9					18.9	0.80	0.56	0.61		28.0	22.0	7.1	8.8
77CW	63.2	52.6					36.8	18.1						29.3								
85AS	63.9	42.6					36.1	57.4														
85AW	100.0	100.0																				
85CS	17.7	15.3	28.8	17.0	22.7	15.3	14.1	28.1	11.0	5.4	5.6	19.0			0.69	0.48	0.10	0.29	29.5	15.0	9.0	8.0
85CW	66.2	57.4					33.8	42.6														

a MBT = (I+Ib+Ic)/(I+Ib+ Ic+II + IIb+ IIc + III), in which IIIb and IIIc are all zero values and not included; CBT = −log[(Ib+IIb)/(I+II)]; T_MBT/CBT_(°C) = (MBT−0.122–0.187^*^CBT)/0.02; pH_CBT_ = (3.33-CBT)/0.38.

All equations are derived from Weijers et al. (2007a).

We also calculated the methylation index of branched tetraethers (MBT) and cyclization ratio of branched tetraethers (CBT), and the temperature and pH estimates derived from them, according to Weijers et al. (2007a) (Table 2). In the hot spring sediment samples, the calculated pH values averaged 7.2 ± 0.4 (*n* = 5) for C-bGDGTs and 7.6 ± 0.4 (*n* = 5) for P-bGDGTs; the former was significantly (*P* < 0.05) lower than the average pH values (8.0 ± 0.6, *n* = 6) calculated from soil C-bGDGTs (Table A1), which is close to the average measured pH value of the soil samples (7.8 ± 0.9, *n* = 6) (Table 1).

The calculated temperatures in sediment samples were statistically indistinguishable (p > 0.2) between the C-bGDGTs (29.3 ± 4.4°C, *n* = 5) and P-bGDGTs (25.0 ± 5.3°C, *n* = 5). The average C-bGDGTs-derived temperature was close to the average measured soil temperature (31.8 ± 1.7°C, *n* = 6) at the time of sampling but was significantly higher than the soil C-bGDGTs-derived temperatures (1.7–22.9°C, average = 11.2 ± 8.0°C, *n* = 6; Table A1), which were on the other hand closer to the mean annual air temperature (MAAT) for the region (10.7°C; Table 1). Schouten et al. (2007) showed that one of the Yellowstone hot springs had MBT and CBT temperatures very close to the local MAAT and suggested that the bGDGTs derived from the surrounding soil area. While we cannot unequivocally exclude the possibility of soil contamination in the hot spring sediment samples in GBS, the MBT-CBT temperatures calculated from the enrichments conducted at high temperature are also in the range of 15–32°C (Table 2), which cannot be explained by soil contamination. Overall, these results suggest that the MBT-CBT proxies derived from soil environments may not be applicable in geothermal environments and that factors other than pH or temperature may control the relative abundance of bGDGTs in geothermal systems.

### Intact polar lipid bGDGTs analyzed by RP-ESI-MS^n^

**Figure A2:**
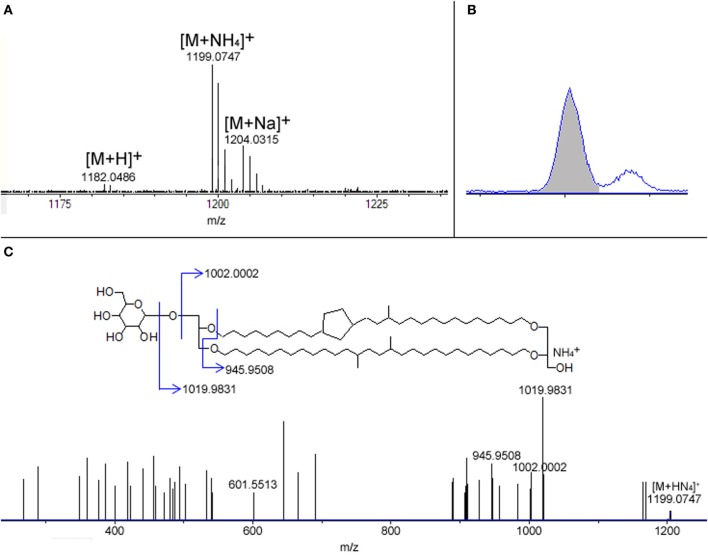
**Occurrence of monoglycosidic bGDGT Ib (1G-bGDGT Ib) at site C in Figure 1, which was determined by RP-ESI-MS^n^. (A)** The measured accurate masses of [M+H]^+^, [M+NH_4_]^+^, [M+Na]^+^ adduct ions. **(B)** Extracted ion chromatogram of 1199.0739 (the theoretical mass of *m/z* [M+NH_4_]^+^) ± 0.05. **(C)** MS^2^ spectra of [M+NH_4_]^+^ adduct ion (Note the ring location is based on Weijers et al., 2006).

Intact glycosidic bGDGTs without hydrolysis have been detected in a peat bog where bGDGT-producing bacteria thrive (Liu et al., 2010). In the GBS, activity of bGDGT-producing bacteria was evidenced by the occurrence of monoglycosidic bGDGT Ib detected using total lipid extract (Figure A2), which accounted for ca. 6.5% of total C-bGDGTs (in terms of total peak areas of [M+Na]^+^, [M+H]^+^, [M+NH_4_]^+^). In contrast, intact polar bGDGTs were undetectable in the adjacent soil samples by RP-ESI-MS^n^, even after purification of soil extracts by preparative HPLC (data not shown), which is consistent with the low P-bGDGTs by the hydrolysis method (above). However, the reverse phase LC-MS method failed to detect other polar bGDGTs obtained by the hydrolysis method (Table 2).

### 16S rRNA gene pyrosequences

Previously published pyrosequencing data (Peacock et al., 2013) further supported the *in situ* production of bGDGTs in the hot spring environment. Peacock et al. (2013) demonstrated that each of the cellulolytic substrates led to enrichment of thermophilic, cellulolytic consortia, as documented by (1) significant increase in DNA yield over un-incubated cellulose substrates, (2) changes in lignocelluloses substrate composition consistent with cellulolysis, and (3) dramatic changes in microbial community composition, with dominant community members closely related to known cellulolytic and hemicellulolytic *Thermotoga* and *Dictyoglomus*, cellulolytic and sugar-fermenting *Desulfurococcales*, and sugar-fermenting and hydrogenotrophic *Archaeoglobales*.

**Figure 4:**
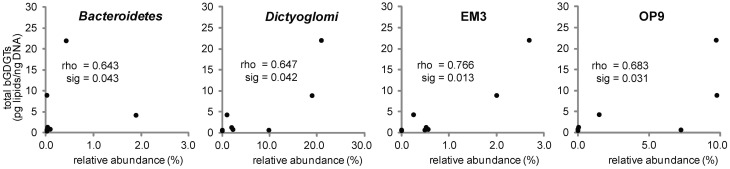
**Spearman's rho correlation coefficients indicated statistically significant, positive relationships between bGDGT absolute abundance and the bacterial Bacteroidetes, Dictyoglomi, EM3, and OP9 (“Atribacteria;” Dodsworth et al., 2013) (1-tailed sig. shown)**. Total bGDGT abundance, including core- and polar lipids, were normalized to ng DNA.

The relative abundance of several bacterial phyla in the pyrotag datasets was significantly correlated with bGDGT abundance: *Bacteroidetes, Dictyoglomi*, and candidate phyla EM3 and OP9 (“Atribacteria” Dodsworth et al., 2013) (Figure 4; Table A2). Within the *Bacteroidetes*, only a single species-level OTU was positively correlated with bGDGT abundance (Table A3); however, that pyrotag could not be assigned to a class or any lower taxonomic level. Similarly, EM3 and OP9 were represented by one and two species-level OTUs that correlated with bGDGT abundance, respectively, but no cultures are available for those candidate phyla (Table A3). Although these groups are plausible sources of bGDGTs, definitive experiments to test whether they are sources of bGDGTs may await isolation and study of representative strains. The phylum *Dictyoglomi* was represented by two OTUs that correlated with bGDGT abundance that could be ascribed to the genus *Dictyoglomus* (Table A3). That genus is currently comprised of only two closely related species, *Dictyoglomus thermophilum* and *D. turdigum*, both of which are obligate fermenters that are known to decompose components of hemicellulose and cellulose (Saiki et al., 1985; Patel et al., 1987). *Dictyoglomus* spp. Rt46-B1 is known to produce predominantly C_16:0_ phospholipid derived fatty acids and traces (<10%) of C_14:0_, C_15:0_, C_17:0_, C_18:0_, C_20:0_, aC_17:0_, C_16:1ω11*c*_, and C_18:1ω9*c*_ (Patel et al., 1991). Our own analysis of lipid extracts from pure cultures of *Dictyoglomus thermophilum* DSM 3960^T^ and *D. turdigum* DSM 6724^T^, both grown on DSMZ 388 medium with gentle agitation in the dark at 70°C for 2 days, failed to reveal bGDGTs (data not shown). This argues against *Dictyoglomi* as a source of bGDGTs, although it is possible that such organisms may produce bGDGTs in the natural environment but not under the laboratory conditions. Lastly, *Acidobacteria* that are commonly believed to be sources of soil/peat bog bGDGTs (Weijers et al., 2006) constituted less than 0.01% of total pyrotags (Peacock et al., 2013) and did not show a significant correlation with bGDGTs in the enrichment, even though the primers used for PCR each match >95% of *Acidobacteria* 16S rRNA gene sequences.

In summary, we show three lines of evidence supporting the *in situ* production of bGDGTs at high temperature in a Great Basin hot spring: (1) the greater abundance of hydrolysis-derived P-bGDGTs over C-bGDGTs in enrichments vs. the opposite in the soil samples and the distinct composition of bGDGTs in the enrichments, (2) the presence of the intact monoglycosidic-bGDGT Ib identified by the RP-ESI-MS^n^ in the hot spring and its absence in the surrounding soil, and (3) the significant correlations between bGDGTs and certain groups of thermophilic bacteria combined with the low abundance of *Acidobacteria*, as inferred from pyrotag data, in all samples.

### Conflict of interest statement

The authors declare that the research was conducted in the absence of any commercial or financial relationships that could be construed as a potential conflict of interest.

## Figures and Tables

**Table A2:**
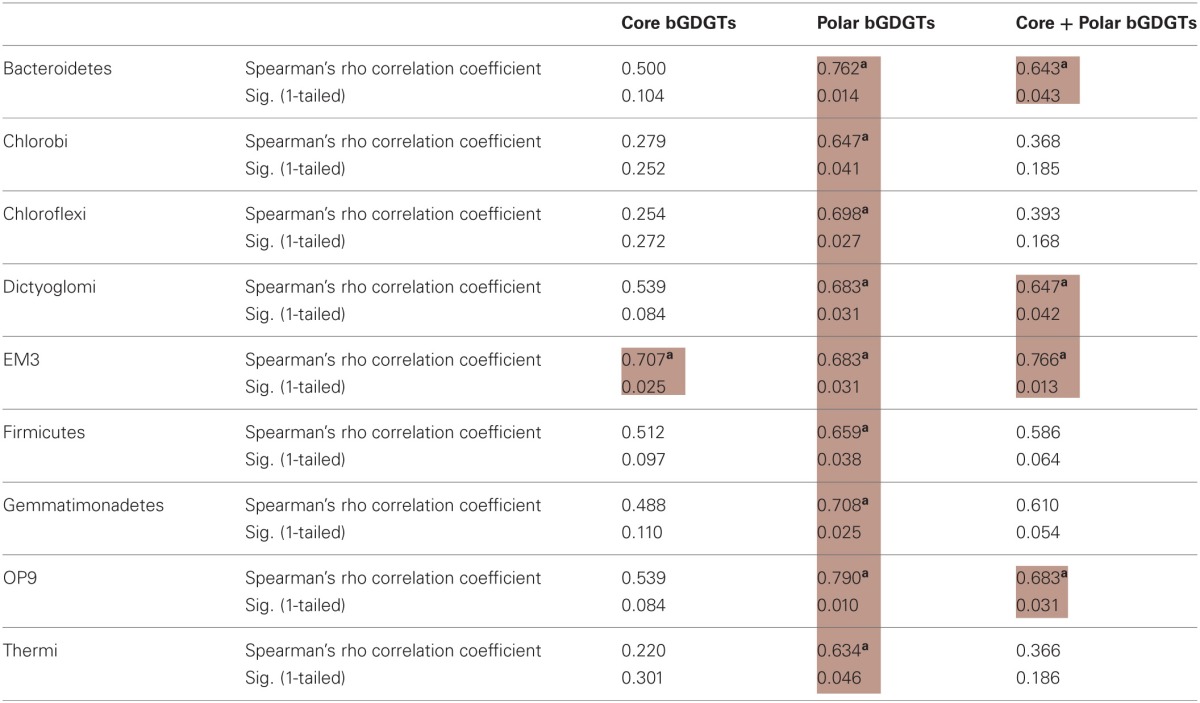
**Correlations between normalized bGDGT concentrations (pg lipids/ng DNA) and relative abundance of bacterial phyla^b^**.

^a^Statistically significant, positive relationships highlighted in pink (1-tailed sig. ≤ 0.05). No archaeal phyla showed significant relationships with bGDGTs.

^b^Relative abundance of phyla-based pyrotag sequence reads.

**Table A3:**
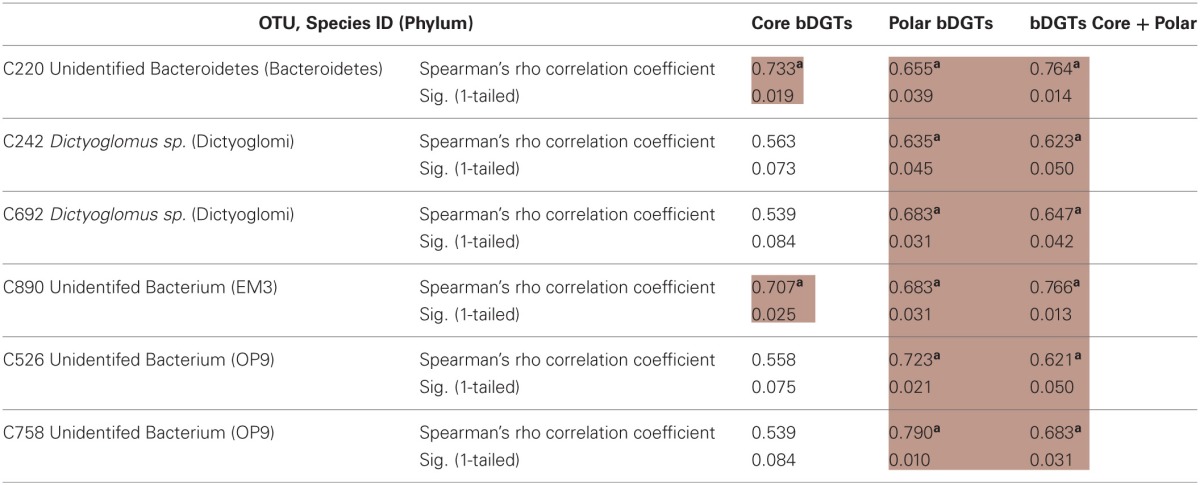
**Correlations between normalized bGDGT concentrations (pg lipids/ng DNA) and relative abundance of dominant bacterial OTUs^b^**.

^a^Statistically significant, positive relationships highlighted in pink (1-tailed sig. ≤ 0.05). No archaeal phyla showed significant relationships with bGDGTs.

^b^Relative abundance of OTUs-based pyrotag sequence reads (See Peacock et al., in review for more detail on OTU assignments).

## Appendix

**Table A1:** **Relative abundances of Core (C)- bGDGTs and C-bGDGTs-derived proxies for soil samples collected along the transect from GBS**.

**Sample**	**Relative abundance of C-bGDGTs (%)**									**C-bGDGT proxies**^**a**^			
	**I**	**Ib**	**Ic**	**II**	**IIb**	**IIc**	**III**	**IIIb**	**IIIc**	**MBT**	**CBT**	**pH_**CBT**_**	**T_**MBT/CBT**_ (°C)**
GBS-10cm	57.7	12.0	2.2	22.5	2.6	0.3	2.6	0.2	0.0	0.72	0.74	6.8	22.9
GBS-20cm	29.8	16.3	1.9	24.9	16.4	1.5	7.2	1.6	0.3	0.48	0.22	8.2	15.8
GBS-30cm	12.1	7.9	1.8	29.0	22.3	1.7	20.2	4.2	0.9	0.22	0.14	8.4	3.5
GBS-50cm	10.7	7.6	1.8	27.8	15.0	1.4	31.7	3.7	0.5	0.2	0.23	8.1	1.7
GBS-150cm	20.9	15.6	8.0	18.8	9.4	0.0	27.3	0.0	0.0	0.44	0.2	8.2	14.3
GBS-300cm	13.7	11.4	6.4	17.1	13.7	0.0	31.5	6.1	0.0	0.32	0.09	8.5	8.9

a MBT = (I+Ib+ Ic)/(I+Ib+ Ic+II + IIb+ IIc + III + IIIb + IIIc).

CBT = −log[(Ib + IIb)/(I + II)]; T_MBT/CBT_ (°C) = (MBT−0.122 − 0.187^*^CBT)/0.02; pH_CBT_ = (3.33–CBT)/0.38. All equations are derived from Weijers et al. (2007a).

## References

[B1] Ballantyne A. P. Greenwood D. R. Sinninghe Damsté J. S. Csank A. Z. Eberle J. J. Rybczynski N. (2010). Significantly warmer Arctic surface temperatures during the Pliocene indicated by multiple independent proxies. Geology 38, 603–606 10.1130/G30815.1

[B2] Bendle J. A. Weijers J. W. H. Maslin M. A. Sinninghe Damsté J. S. Schouten S. Hopmans E. C. (2010). Major changes in glacial and Holocene terrestrial temperatures and sources of organic carbon recorded in the Amazon fan by tetraether lipids. Geochem. Geophys. Geosyst. 11:Q12007 10.1029/2010GC003308

[B3] Cole J. K. Peacock J. P. Dodsworth J. A. Williams A. J. Thompson D. B. Dong H. (2013). Sediment microbial communities in Great Boiling Spring, Nevada, are controlled by temperature and distinct from planktonic communities. ISME J. 7, 718–729 10.1038/ismej.2012.157 23235293 PMC3605714

[B4] Costa K. C. Navarro J. B. Shock E. L. Zhang C. L. Soukup D. Hedlund B. P. (2009). Microbiology and geochemistry of Great Boiling and Mud Hot Springs in the United States Great Basin. Extremophiles 13, 447–459 10.1007/s00792-009-0230-x 19247786

[B5] DeRosa M. Gambacorta A. Huber R. Lanzotti V. Nicolaus B. Stetter K. O. (1988). A new 15,16-dimethyl-30-glyceryloxytriacontanoic acid from lipids of *Thermotoga maritima*. J. Chem. Soc. Chem. Commun. 1988, 1300–1301 10.1039/c39880001300

[B6] Dodsworth J. A. Hungate B. A. Hedlund B. P. (2011). Ammonia oxidation, denitrification and dissimilatory nitrate reduction to ammonium in two US Great Basin hot springs with abundant ammonia-oxidizing archaea. Environ. Microbiol. 8, 2371–2386 10.1111/j.1462-2920.2011.02508.x 21631688

[B7] Dodsworth J. A. Blainey P. C. Murugapiran S. K. Swingley W. D. Ross C. A. Tringe S. G. (2013). Single-cell and metagenomic analyses indicate a fermentative, saccharolytic lifestyle for members of the OP9 lineage. Nat. Commun. 4:1854 10.1038/ncomms2884 23673639 PMC3878185

[B8] Fawcett P. J. Werne J. P. Anderson R. S. Heikoop J. M. Brown E. T. Berke M. A. (2011). Extended megadroughts in the southwestern United States during Pleistocene interglacials. Nature 470, 518–521 10.1038/nature09839 21350483

[B9] Hopmans E. C. Schouten S. Pancost R. D. Van der Meer M. T. J. Sinninghe Damsté J. S. (2000). Analysis of intact tetraether lipids in archaeal cell material and sediments by high performance liquid chromatography/atmospheric pressure chemical ionization mass spectrometry. Rapid Commun. Mass Spectrom. 14, 585–589 10775092 10.1002/(SICI)1097-0231(20000415)14:7<585::AID-RCM913>3.0.CO;2-N

[B10] Hopmans E. C. Weijers J. W. H. Schefuss E. Herfort L. Sinninghe Damsté J. S. Schouten S. (2004). A novel proxy for terrestrial organic matter in sediments based on branched and isoprenoid tetraether lipids. Earth Planet. Sci. Lett. 224, 107–116 10.1016/j.epsl.2004.05.012

[B11] Hu J. Meyers P. A. Chen G. Peng P. Yang Q. (2012). Archaeal and bacterial glycerol dialkyl glycerol tetraethers in sediments from the Eastern Lau Spreading Center, South Pacific Ocean. Org. Geochem. 43, 162–167 10.1016/j.orggeochem.2011.10.012

[B12] Huang Z. Wiegel J. Zhou J. Hedlund B. Zhang C. L. (2007). Molecular phylogeny of uncultivated *Crenarchaeota* in Great Basin hot springs of moderately elevated temperature. Geomicrobiol. J. 24, 535–542 10.1080/01490450701572523

[B13] Huber R. Wilharm T. Huber D. Trincone A. Burggraf S. König H. (1992). *Aquifex pyrophilus* gen. nov. sp. nov., represents a novel group of marine hyperthermophilic hydrogen-oxidizing bacteria. Syst. Appl. Microbiol. 15, 340–351 10.1016/S0723-2020(11)80206-7

[B14] Huber R. Rossnagel P. Woese C. R. Rachel R. Langworthy T. A. Stetter K. O. (1996). Formation of ammonium from nitrate during chemolithoautotrophic growth of the extremely thermophilic bacterium *Ammonifex degensii* gen.nov.sp.nov. Syst. Appl. Microbiol. 19, 40–49 10.1016/S0723-2020(96)80007-5 11539844

[B16] Kim J. H. Zarzycka B. Buscail R. Peters F. Bonnin J. Ludwig W. (2010). Contribution of river−borne soil organic carbon to the Gulf of Lions (NW Mediterranean). Limnol. Oceanogr. 55, 507–518 10.4319/lo.2009.55.2.0507

[B17] Kim J. H. Zell C. Moreira-Turcq P. Perez M. A. P. Abril G. Mortillaro J. M. (2012). Tracing soil organic carbon in the lower Amazon River and its tributaries using GDGT distributions and bulk organic matter properties. Geochim. Cosmochim. Acta 90, 163–180 10.1016/j.gca.2012.05.014

[B18] Langworthy T. A. Holzer G. Zeikus J. G. Tornabene T. G. (1983). Iso- and anteiso-branched glycerol diethers of the thermophilic anaerobe *Thermodesulfotobacterium commune*. Syst. Appl. Microbiol. 4, 1–17 10.1016/S0723-2020(83)80029-0 23196295

[B18a] Lincoln S. A. Bradldy A. S. Newman S. A. Summons R. E. (2013). Archaeal and bacterial glycerol dialkyl glycerol tetraether lipids in chimneys of the Lost City Hydrothermal Field. Org. Geochem. 60, 45–53 10.1016/j.orggeochem.2013.04.010

[B19] Liu X.-L. Leider A. Gillespie A. Gröger J. Versteegh G. J. M. Hinrichs K-U. (2010). Identification of polar lipid precursors of the ubiquitous branched GDGT orphan lipids in a peat bog in Northern Germany. Org. Geochem. 41, 653–660 10.1016/j.orggeochem.2010.04.004

[B20] Liu X.-L. Summons R. E. Hinrichs K.-U. (2012). Extending the known range of glycerol ether lipids in the environment: structural assignments based on MS/MS fragmentation patterns. Rap. Commun. Mass Spectrom. 26, 2295–2302 10.1002/rcm.6355 22956321

[B21] Patel B. K. C. Morgan H. W. Daniel R. M. (1987). Isolation of an extremely thermophilic chemoorganotrophic anaerobe similar to *Dictyoglomus thermophilum* from a New Zealand hot spring. Arch. Microbiol. 147, 21–24 10.1007/BF00492899

[B22] Patel B. K. C. Skerratt J. H. Nichols P. D. (1991). The phospholipid ester-linked fatty acid composition of thermophilic bacteria. Syst. Appl. Microbiol. 14, 311–316 10.1016/S0723-2020(11)80304-8

[B23] Peacock J. P. Cole J. K. Murugapiran S. K. Dodsworth J. A. Fisher J. C. Moser D. P. (2013). Pyrosequencing reveals high-temperature cellulolytic microbial consortia in Great Boiling Spring after *in situ* lignocellulose enrichment. PLoS ONE 8:e59927 10.1371/journal.pone.0059927 23555835 PMC3612082

[B24] Pearson A. Huang Z. Ingalls A. E. Romanek C. S. Wiegel J. Freeman K. H. (2004). Nonmarine crenarchaeol in Nevada hot springs. Appl. Environ. Microbiol. 70, 5229–5237 10.1128/AEM.70.9.5229-5237.2004 15345404 PMC520871

[B25] Pearson A. Pi Y. Zhao W. Li W. Li Y. Inskeep W. (2008). Factors controlling the distribution of archaeal tetraethers in terrestrial hot springs. Appl. Environ. Microbiol. 74, 3523–3532 10.1128/AEM.02450-07 18390673 PMC2423032

[B26] Peterse F. Schouten S. van der Meer J. van der Meer M. T. J. Sinninghe Damsté J. S. (2009). Distribution of branched tetraether lipids in geothermally heated soils: implications for the MBT/CBT temperature proxy. Org. Geochem. 40, 201–205 10.1016/j.orggeochem.2008.10.010

[B27] Peterse F. Nicol G. W. Schouten S. Sinninghe Damsté J. S. (2010). Influence of soil pH on the abundance and distribution of core and intact polar lipid-derived branched GDGTs in soil. Org. Geochem. 41, 1171–1175 10.1016/j.orggeochem.2010.07.004

[B28] Peterse F. Prins M. A. Beets C. J. Troelstra S. R. Zheng H. Gu Z. (2011). Decoupled warming and monsoon precipitation in East Asia over the last deglaciation. Earth Planet. Sci. Lett. 301, 256–264 10.1016/j.epsl.2010.11.010

[B29] Peterse F. van der Meer J. Schouten S. Weijers W. H. Fierer N. Jackson R. B. (2012). Revised calibration of the MBT–CBT paleotemperature proxy based on branched tetraether membrane lipids in surface soils. Geochim. Cosmochim. Acta 96, 215–229 10.1016/j.gca.2012.08.011

[B30] Rueda G. Rosell-Melé A. Escala M. Gyllencreutz R. Backman J. (2009). Comparison of instrumental and GDGT-based estimates of sea surface and air temperatures from the Skagerrak. Org. Geochem. 40, 287–291 10.1016/j.orggeochem.2008.10.012

[B31] Saiki T. Kobayashi Y. Kawagoe K. Beppu T. (1985). *Dictyoglomus thermophilum* gen. nov., sp. nov., a chemoorganotrophic, anaerobic, thermophilic bacterium. Int. J. Syst. Bacteriol. 35, 253–259 10.1099/00207713-35-3-253

[B32] Schouten S. Hopmans E. C. Pancost R. D. Sinninghe Damsté J. S. (2000). Widespread occurrence of structurally diverse tetraether membrane lipids: evidence for the ubiquitous presence of low-temperature relatives of hyperthermophiles. Proc. Natl Acad. Sci. U.S.A. 97, 14421–14426 10.1073/pnas.97.26.14421 11121044 PMC18934

[B33] Schouten S. van der Meer M. T. J. Hopmans E. C. Rijpstra W. I. C. Reysenbach A.-L. Ward D. M. (2007). Archaeal and bacterial glycerol dialkyl glycerol tetraether lipids in hot springs of Yellowstone National Park. Appl. Environ. Microbiol. 73, 6181–6191 10.1128/AEM.00630-07 17693566 PMC2074994

[B34] Schouten S. Eldrett J. Greenwood D. R. Harding I. Baas M. Sinninghe Damsté J. S. (2008). Onset of long-term cooling of Greenland near the Eocene–Oligocene boundary as revealed by branched tetraether lipids. Geology 36, 147–150 10.1130/G24332A.1

[B35] Schouten S. Hopmans E. C. Sinninghe Damsté J. S. (2013). The organic geochemistry of glycerol dialkyl glycerol tetraether lipids: a review. Org. Geochem. 54, 19–61 10.1016/j.orggeochem.2012.09.006

[B36] Sinninghe Damsté J. S. Ossebaar J. Abbas B. Schouten S. Verschuren D. (2009). Fluxes and distribution of tetraether lipids in an equatorial African lake: constraints on the application of the TEX_86_ palaeothermometer and BIT index in lacustrine settings. Geochim. Cosmochim. Acta 73, 4232–4249 10.1016/j.gca.2009.04.022

[B37] Sinninghe Damsté J. S. Rijpstra W. I. C. Hopmans E. C. Weijers W. H. Foesel B. U. Overmann J. (2011). 13,16-dimethyl octacosanedioic acid (*iso*-diabolic acid), a common membrane-spanning lipid of *Acidobacteria* subdivisions 1 and 3. Appl. Environ. Microbiol. 77, 4147–4154 10.1128/AEM.00466-11 21515715 PMC3131667

[B38] Sturt H. F. Summons R. E. Smith K. Elvert M. Hinrichs K.-U. (2004). Intact polar membrane lipids in prokaryotes and sediments deciphered by high-performance liquid chromatography/electrospray ionization multistage mass spectrometry—new biomarkers for biogeochemistry and microbial ecology. Rapid Commun. Mass Spectrom. 18, 617–628 10.1002/rcm.1378 15052572

[B39] Sun Q. Chu G. Liu M. Xie M. Li S. Ling Y. (2011). Distributions and temperature dependence of branched glycerol dialkyl glycerol tetraethers in recent lacustrine sediments from China and Nepal. J. Geophys. Res. 116:G01008

[B40] Tierney J. E. Russell J. M. (2009). Distributions of branched GDGTs in a tropical lake system: implications for lacustrine application of the MBT/CBT paleoproxy. Org. Geochem. 40, 1032–1036 10.1016/j.orggeochem.2009.04.014

[B41] Tierney J. E. Schouten S. Pitcher A. Hopmans E. C. Sinninghe Damsté J. S. (2011). Core and intact polar glycerol dialkyl glycerol tetraethers (GDGTs) in Sand Pond, Warwick, Rhode Island (USA): Insights into the origin of lacustrine GDGTs. Geochim. Cosmochim. Acta 15, 561–581

[B42] Tyler J. J. Nederbragt A. J. Jones V. J. Thurow J. W. (2010). Assessing past temperature and soil pH estimates from bacterial tetraether membrane lipids: evidence from the recent lake sediments of Lochnagar, Scotland. J. Geophys. Res. 115:G01015 10.1029/2009JG001109

[B43] Wang H. Liu W. Zhang C. L. Wang Z. Wang J. Liu Z. (2012). Distribution of glycerol dialkyl glycerol tetraethers in surface sediments of Lake Qinghai and surrounding soils. Org. Geochem. 47, 78–87 10.1016/j.orggeochem.2012.03.008

[B44] Weijers J. W. H. Schouten S. Hopmans E. C. Geenevasen J. A. J. David O. R. P. Coleman J. M. (2006). Membrane lipids of mesophilic anaerobic bacteria thriving in peats have typical archaeal traits. Environ. Microbiol. 8, 648–657 10.1111/j.1462-2920.2005.00941.x 16584476

[B45] Weijers J. W. H. Schouten S. van den Donker J. C. Hopmans E. C. Sinninghe Damsté J. S. (2007a). Environmental controls on bacterial tetraether membrane lipid distribution in soils. Geochim. Cosmochim. Acta 71, 703–713 10.1016/j.gca.2006.10.003

[B46] Weijers J. W. H. Schefuβ E. Schouten S. Sinninghe Damsté J. S. (2007b). Coupled thermal and hydrological evolution of tropical Africa over the last deglaciation. Science 315, 1701–1704 10.1126/science.1138131 17379805

[B47] Weijers J. W. H. Schouten S. Sluijs A. Brinkhuis H. Sinninghe Damsté J. S. (2007c). Warm Arctic continents during the Palaeocene–Eocene thermal maximum. Earth Planet. Sci. Lett. 261, 230–238 10.1016/j.epsl.2007.06.033

[B48] Weijers J. W. H. Bernhardt B. Peterse F. Werne J. P. Dungait J. A. J. Schouten S. (2011). Absence of seasonal patterns in MBT-CBT indices in mid-latitude soils. Geochim. Cosmochim. Acta 75, 3179–3190 10.1016/j.gca.2011.03.015

[B49] Yang G. Zhang C. L. Xie S. Gao M. Ge Z. Yang Z. (2013). Microbial glycerol dialkyl glycerol tetraethers from river water and soil near the Three Gorges Dam on the Yangtze River. Org. Geochem. 56, 40–50 10.1016/j.orggeochem.2012.11.014

[B50] Zhang C. L. Pearson A. Li Y.-L. Mills G. Wiegel J. (2006). A thermophilic temperature optimum for crenarchaeol and its implication for archaeal evolution. Appl. Environ. Microbiol. 72, 4419–4422 10.1128/AEM.00191-06 16751559 PMC1489640

[B51] Zhang C. L. Huang Z. Li Y.-L. Romanek C. S. Mills G. Wiegel J. (2007). Lipid biomarkers, carbon isotopes, and phylogenetic characterization of bacteria in California and Nevada hot springs. Geomicrobiol. J. 24, 519–534 10.1080/01490450701572515

[B52] Zhang C. L. Ye Q. Huang Z. Li W. Chen J. Song Z. Hedlund B. P. (2008). Global occurrence of putative archaeal *amoA* genes from terrestrial hot springs. Appl. Environ. Microbiol. 74, 6417–6426 10.1128/AEM.00843-08 18676703 PMC2570307

[B53] Zhang C. L. Wang J. Wei Y. Zhu C. Huang L. Dong H. (2012). Production of branched tetraether lipids in the lower Pearl River and estuary: effects of extraction methods and impact on bGDGT proxies. Front. Terr. Microbiol. 2, 1–18 10.3389/fmicb.2011.00274 22291686 PMC3253547

[B54] Zhou H. Hu J. Ming L. Peng P. Zhang G. (2011). Branched glycerol dialkyl glycerol tetraethers and paleoenvironmental reconstruction in Zoigê peat sediments during the last 150 years. Chinese Sci. Bull. 56, 2456–2463 10.1007/s11434-011-4594-9

[B55] Zhu C. Weijers J. W. H. Wagner T. Pan J-M. Chen J-F. Pancost R. D. (2011). Sources and distributions of tetraether lipids in surface sediments across a large river-dominated continental margin. Org. Geochem. 4, 376–386 10.1016/j.orggeochem.2011.02.002

